# Fortified edible oils in Bangladesh: A study on vitamin A fortification and physicochemical properties

**DOI:** 10.1016/j.heliyon.2024.e25489

**Published:** 2024-02-03

**Authors:** Rokeya Begum, MdRakibul Hasan, Shamoli Akter, MdNannur Rahman

**Affiliations:** Department of Food Technology and Nutritional Science, Mawlana Bhashani Science and Technology University, Santosh, Tangail-1902, Bangladesh

**Keywords:** Fortified edible oil, Vitamin A, Chemical parameters, Physical parameters, Peroxide value

## Abstract

Food fortification has always been an effective and proven practice for eradicating various nutrient deficiencies in Bangladesh. This study investigated different quality parameters of three types (soybean, sunflower, and palm) of extensively consumed fortified edible oils in Bangladesh. Vitamin A analysis has shown that the vitamin A fortification level of most of the oil brands (73 %) did not comply with the Bangladesh Standard and Testing Institution (BSTI) standards (1.5–3.0 mg/100 g). Vitamin A contents of soybean, sunflower, and palm oil brands ranged from 0.13 to 2.06, 0.92–1.34, and 0.99–1.31 mg/100 g, respectively. Inter-brand values of vitamin A were also significantly different (*p* < 0.05). The majority of the samples were found to be within the acceptable ranges of Codex and BSTI, taking into account the significant chemical quality parameters for soybean, sunflower, and palm oil, such as acid value (0.31–0.93, 0.31–0.56, 0.39–0.81 mg KOH/g), free fatty acid (0.15–0.46, 0.15–0.28, 0.2–0.41 %), saponification (188.64–196.35, 186.53–188, 197.05–199.86 mg KOH/g), and peroxide values (0.06–2.9, 0.65–1.58, 1.35–1.75 meq O_2_/kg) respectively. All the brands' physical quality parameters (density, specific gravity, pH, viscosity, smoke point, color, and RI) complied with Codex standards. Various physical and chemical quality parameters were analyzed for significant correlations at 0.01 and 0.05 levels of significance. Remarkably, significant correlations were found between vitamin A and peroxide value (*p* < 0.01), iodine value and viscosity (*p* < 0.01), saponification value and viscosity (*p* < 0.01), pH and viscosity (*p* < 0.01), and saponification value and pH (*p* < 0.05). In conclusion, although the vitamin A status of most of the fortified edible oil brands was poor, the key quality indicators (except iodine value) of most of the oils were within the Codex and BSTI standard limits and were acceptable for human consumption.

## 1 Introduction

Food fortification in lower-middle-income countries effectively addresses micronutrient deficiencies [1]. Large-scale food fortification programs significantly influence nutritional outcomes, including the worldwide alleviation of vitamin A, iron, iodine, and folic acid deficiencies [2]. In Bangladesh, several fortified food items are available and largely consumed. Among the edible oils, soybean oil has emerged as a prime contender for viable vitamin A fortification because of its centralized processing, extensive supply, and high consumption rate [3]. Approximately 99 percent of the Bangladeshi population consumes vegetable oil daily [4]. Bangladeshis consume the most of the two edible oils that are made from soybeans and palms, especially super palm oil [5]. Though the Bangladesh Government passed the National Edible Oil Fortification ‘Law’ in 2013, it has been fortifying edible oils with vitamin A since 2011 [6]. Vitamin A deficiency remains a public health concern despite Bangladesh's higher consumption rate of edible oils fortified with vitamin A. According to the reports of the Bangladesh Bureau of Statistics (BBS) and UNICEF Bangladesh [7], about 21 % of Bangladesh's preschoolers and 5 % of its women suffer from vitamin A deficiency. Therefore, assessing the vitamin A content in fortified edible oils is time demanding. Fortified edible oils are consumed widely; thus, the quality should be uncompromised and up to standards. The main attributes of edible oils can be assessed by looking at their acid, peroxide, and iodine values, which show how much the oil has been hydrolyzed and oxidized and how much unsaturated fatty acid it has [8]. A decline in key quality parameters, including acid content, peroxide levels, iodine value, etc., has the potential to diminish the overall quality of the oil [9]. For example, the deterioration of added vitamin A in the oil occurs due to the formation of peroxides during the oil production or distribution process. This leads to undesirable changes in the oil's quality, such as rancidity, posing a threat to the intended objectives of oil fortification [10]. Similarly, the iodine value, an authentication tool for measuring the degree of fatty acid unsaturation in edible oils, can be used to identify inexpensive adulterated vegetable oil with a lot of saturated fatty acids [11]. The Bangladesh Standard and Testing Institution (BSTI) has established standards that align with the Codex standards. These standards ensure the production quality of edible fortified oils. But there is scarce data regarding the fortified oil's quality. Hence, it has become very important to know the vitamin A fortification status and other quality parameters of existing fortified edible oils in Bangladesh, which will provide adequate information on fortified oil quality to the manufacturer, consumers, and other concerned stakeholders. This study will objectively evaluate vitamin A and other physicochemical quality indicator contents of fortified edible oils in Bangladesh.

## 2 Materials and methods

### 2.1 Sample collection and storage

A total of 13 packaged oil brands, comprising 9 soybean, 3 sunflower, and 1 palm oil brand were purchased from local marketplaces of Dhaka city, Bangladesh. Additionally, two varieties of open bulk oil (palm and soybean) were obtained, resulting in a total of 45 samples, with three samples collected from each brand, all representing the latest production batches. Each sample was given a special sample ID number on the label to assure sample identification (Table 1). All the samples were collected within January 2023. After collection the samples were kept in black plastic bags in a cardboard box to protect them from sunlight, prevent contamination, and ensure good storage conditions until the analysis. Samples were maintained at room temperature (25 ± 5 °C) in the dark.

**Table 1:** Coding of fortified edible oil samples.

Samples	Code
Soybean brand 1	SB1
Soybean brand 2	SB2
Soybean brand 3	SB3
Soybean brand 4	SB4
Soybean brand 5	SB5
Soybean brand 6	SB6
Soybean brand 7	SB7
Soybean brand 8	SB8
Soybean brand 9	SB9
Bulk soybean oil	SB10
Sunflower brand 1	SF1
Sunflower brand 2	SF2
Palm oil brand 1	PM1
Bulk palm oil	PM2

### 2.2 Reagents and chemicals

For vitamin A analysis, Retinyl palmitate (Vitamin A) standard (R3375-14, Type IV, ∼1,800,000 USP units/g, Sigma-Aldrich, Germany), HPLC grade n-heptane, 99+% (A Johnson Matthey Company, UK), and HPLC grade 2-propanol, ≥99.9 % (Merck KGaA, Germany) were purchased after checking highest quality. All the chemical tests were conducted using highly pured chemicals and reagents including hydrochloric acid, ethanol (99 %), potassium hydroxide, phenolphthalein indicator, acetic acid, chloroform, sodium thiosulphate, starch solution, Wijs iodine solution, potassium iodide solution, phloroglucinol solution.

### 2.3 Vitamin a analysis

#### 2.3.1 High-performance liquid chromatography

A normal-phase Shimadzu LC-20 HPLC system (Shimadzu Corporation, Japan) was used to analyze vitamin A using the method described by Hasan et al. [12]. The system included a UV detector (SPD-20A/20AV), two high-pressure pumps of series-type double plunger (Model: LC-20AT), and data analysis software (Lab Solution LC/GC; version 5.30 SPI). The mobile phase, composed of 0.2 % isopropyl alcohol in n-heptane and 100 % n-heptane in a ratio of 25:75 (v/v), was used with a “Restek” Pinnacle DB silica (250 × 4.6 mm; 5 μm particle size) column (USA), at a flow rate of 1 mL/min and a 20 μL injector loop (for Shimadzu LC-6A HPLC system). A 10 min post-run for the system equilibration was used. Vitamin A was detected at a 326 nm wavelength.

#### 2.3.2 Sample preparation

One drop of fortified edible oil sample (∼1.00 mg) was weighed by a digital balance machine and taken into a 10 mL volumetric flask. HPLC grade n-heptane was then added to the sample for up to 10 mL. The volumetric flask was labeled with sample concentration (e.g., mg/mL). The sample solution was vortexed (TRXH-C, Oscillator, China) for 5 min, followed by sonication (Digital Ultrasonic Bath, UBT-580) for another 5 min. After mixing the sample and n-heptane, the sample solution was filtered with a PTEF syringe filter (0.45 μM) using a 5 mL disposable syringe. The filtered sample was transferred in a microcentrifuge and injected to HPLC system.

### 2.4 Determination of chemical properties

#### 2.4.1 Acid value

The acid value of edible oils was determined by titrimetric method according to AOAC [13] and free fatty acid was determined using a mathematical expression. Approximately 5 g of oils were mixed with 25 ml of neutral ethyl alcohol, and the mixture was heated on a water bath. While hot, 1 ml of phenolphthalein indicator solution was added. The hot mixture was titrated against 0.1 N alcoholic potassium hydroxide solution with vigorous shaking. Acid value and free fatty acid was calculated using the following equations number (1) and (2) respectively.

(1) $$\text{Acid}\;\text{value}=\frac{V\times N\times 56.1}{W}$$

(2) $$\text{Free}\;\text{fatty}\;\text{acid}=\frac{\text{Acid}\;\text{value}}{2}$$

Where, V = volume of standard KOH solution in ml; N = normality of standard KOH solution; W = weight of oil sample in grams; 56.1 = Equivalent weight of potassium hydroxide.

#### 2.4.2 Peroxide value

The peroxide value of edible oils was determined by titrimetric method according to AOAC, Official Method [14]. 5 g of oil sample was mixed with 30 mL mixture of acetic acid and chloroform (3:2) and was swirled for dissolution. 1 mL of potassium iodide solution was added, and the solution stood for 1 min in darkness with occasional shaking. 30 mL of distilled water was added. The liberated iodine was titrated with 0.01 N sodium thiosulphate until the yellow color disappeared, followed by the addition of 1 mL starch solution indicator. Vigorous shaking continued to release all I2 from the CH_3_Cl layer until the blue color vanished. The peroxide value was estimated using the following equation number (3):

(3) Peroxidevalue=(V×N×100)/W

Where, Where V = volume of sodium thiosulphate, N = normality used for titre, and W = weight of the sample.

#### 2.4.3 Iodine value and refractive index

The iodine value of edible oils was determined according to AOCS, Official Method [15] where 0.5 g of edible oil was measured and mixed with 10 ml of carbon tetrachloride and 25 ml of Wijs solution. A blank determination was conducted alongside the sample. The solutions were then stored in a dark cupboard for 30 min. Afterward, 15 ml of 10 % potassium iodide solution and 100 ml of distilled water were added to both flasks. A few drops of starch indicator were introduced, and titration with 0.1 N Na_2_S_2_O_3_ solution commenced. The titration continued until the yellow solution turned almost colorless, and further titration was performed until the blue color entirely disappeared. Iodine value was calculated by the following formula number (4):

(4) Iodinevalue(IV)=[(B−A)×N×0.127×100]/W

Where, B = ml of 0.1 N Na_2_S_2_O_3_ required by blank, A = ml of 0.1 N Na_2_S_2_O_3_ required by oil sample, N = normality of Na_2_S_2_O_3_, W = weight of oil in g, 1 ml 1 N Na_2_S_2_O_3_ = 0.127 g I_2_

Refractive index was determined using following mathematical equation number (5) which was derived by Perkins [16].

(5) Refractiveindex=1.45765+0.0001164IV

#### 2.4.4 Saponification value & glycerin (%)

The saponification value of edible oils was determined according to AOAC, Official Method [17] and glycerin % were derived from mathematical expressions. Where two grams of oil samples were mixed with 25 mL of 0.5 N alcoholic potassium hydroxide solution. Blank determination was conducted simultaneously. The flasks were covered with aluminum foil and gently boiled in a hot water bath at 65 °C for 1 h until saponification was complete. After cooling, 1 mL of phenolphthalein indicator was added, and excess potassium hydroxide was titrated with 0.5 N hydrochloric acid until the pink color disappeared. The saponification value and glycerine (%) were estimated using the following equations number (6) and (7) respectively:

(6) Saponificationvalue=[56.1×(B−A)×N]/W

(7) Glycerine(%)=(Saponificationvalue−Acidvalue)×0.054664

Where, W is weight of sample, B is blank titre value, A is sample titre value, and N is 0.5 normality of HCl.

#### 2.4.5 Qualitative test for rancidity in oil

The rancidity of the oil samples was determined qualitatively using Kries test as described by Pearson [18] where, 5 ml of the oil sample was vigorously mixed with 5 ml of 0.1 % phloroglucinol solution in diethyl ether. 5 ml of concentrated Hydrochloric acid was added and mixed for about 20s. The presence of pink color indicates incipient rancidity.

### 2.5 Determination of physical properties

#### 2.5.1 Color analysis

Color evaluation of fortified edible oil samples was conducted by measuring Hunter L, a*, and b* values using a Chroma meter (CR-400; Konica-Minolta, Tokyo, Japan) according to Rhim and Hong [19]. The instrument, calibrated with a standard white tile, assessed lightness (L), red/green value (a*), and yellow/blue value (b*). Measurements were taken at three different spots on transparent mini liquid holders made of polypropylene, with at least three readings each.

#### 2.5.2 Density and specific gravity

The density and specific gravity of edible oil were determined using a dry pycnometer according to Kumar et al. [20]. Density was assessed as the mass of the oil sample per unit volume, while specific gravity was measured as the ratio of oil density to the density of distilled water at the same temperature. Distilled water was added to pycnometers, and measurements were taken with a digital balance for both water and oil weights. The density and specific gravity were calculated using the following equations number (8) and (9) respectively:

(8) $$\text{Density}=\frac{\text{Mass}\;\text{of}\;\text{the}\;\text{oil}\;\left(g\right)}{\text{Volume}\;\text{of}\;\text{the}\;\text{oil}\;\left(\text{cm}3\right)}$$

(9) $$\text{Specific}\;\text{gravity}=\frac{\text{Density}\;\text{of}\;\text{oil}}{\text{Density}\;\text{of}\;\text{water}}$$

#### 2.5.3 Odor analysis

The odor of the oil samples was determined using a glass stoppered bottle rinsed with 4 M HCl internally and externally and rinsed with distilled water. The bottle was halfway filled with the oil sample and shake vigorously for about 2 min. The stopper was then removed and the odor was observed by putting nostrils near the mouth of the bottle and odor was reported as either offensive or inoffensive [21].

#### 2.5.4 Smoke point

Smoke point of the edible oil samples was determined according to Sarwar et al. [22]. Approximately 100 ml of the oil sample was placed in a clean beaker and heated on a plate heater until visible smoke emerged. The smoke point was recorded using a digital meat thermometer (LCD Multi-Thermometer: 50 °C to +300 °C).

#### 2.5.5 pH

The pH of edible oil samples was determined by measuring the concentration of hydrogen ions. Using a digital pH meter (STARTER 3100), calibration was performed with pH 4.1, 7, and 10 standard solutions prior to the actual measurements, following the protocol by Kumar et al. [20]. Approximately 50 cm^3^ of the oil sample was placed in a beaker, and the pH meter electrode was immersed for digital recording.

#### 2.5.6 Viscosity

The Lab Viscometer Viscosity Tester (DVS-80S, Occulab) was employed to determine the dynamic viscosity of the edible oils [23]. Rotor 1 (L = 6.2 cm, d = 1.5 cm) was utilized for viscosity measurements at 60 RPM, and the spindle rotated in the oil for 1 min until the meter's reading stabilized. Three readings were taken for each sample.

### 2.6 Statistical analysis

Statistical Package for the Social Sciences (SPSS version 20.0 SPSS Inc. Chicago, USA) was used for data analysis. The mean values were always followed by the standard deviations to show the differences between measurements of the parameters. The significance of the findings was assessed using a one-way ANOVA test. The graphical representations of the parameters of the edible oils were generated using Microsoft Excel version 10.0. A 95 % confidence interval was used with a significance level of 0.05.

## 3 Results and discussion

### 3.1 Vitamin a content of fortified edible oils

**Fig. 1:**
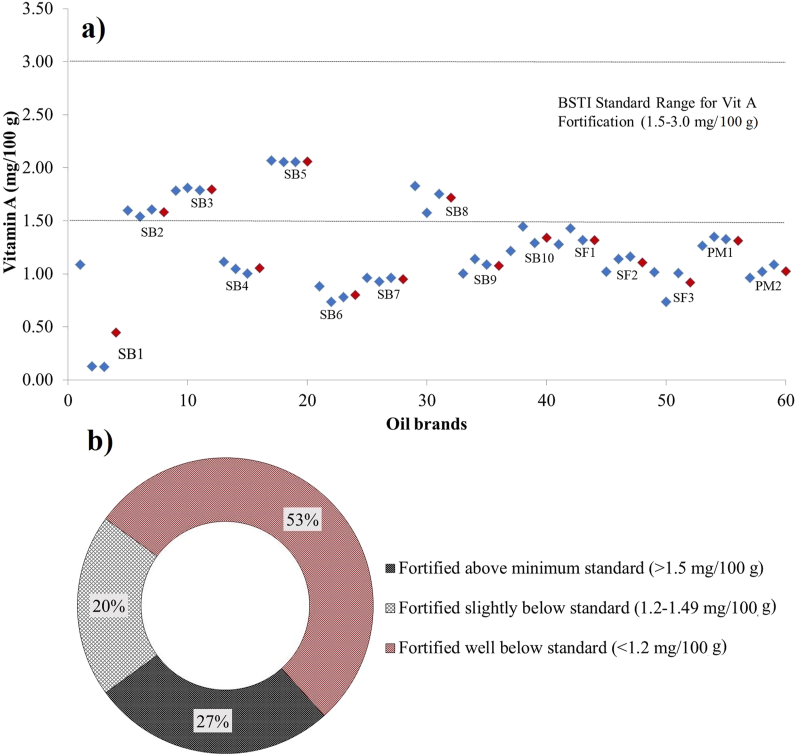
a) Vitamin A content of fortified edible oils; b) Percentage of vitamin A fortification status of oil brands; SB: Soybean; SF: Sunflower; PM: Palm Oil; The mean value for a brand is displayed in red, and the mean values for three replicas are displayed in blue. (For interpretation of the references to color in this figure legend, the reader is referred to the Web version of this article.)

Fig. 1 depicted the vitamin A content of fortified edible oil brands (soybean, sunflower and palm oils). The fortification status of all the oil samples in Fig. 1 (a) showed that all the brands, including bulk soybean oil and palm oil, were vitamin A fortified. Among the soybean oil samples, only 4 brands (SB2, SB3, SB5, and SB8) had vitamin A above the minimum recommended BSTI standard (1.5 mg/100 g) [24].

Among all the edible oil samples highest and lowest vitamin A content was found in soybean oil samples ranging from 2.09 to 0.13 mg/100 g. Whereas the sunflower and palm oils have vitamin A below the minimum standards of BSTI ranged from 1.34 to 0.92 mg/100 g and 1.31–0.99 mg/100 g respectively [Fig. 1 (a)]. Among the soybean oil samples the highest and lowest vitamin A contents were found in SB5 (2.09 mg/100 g) and SB1 (0.13 mg/100 g), which are significantly different (*p* < 0.05). Remarkably the bulk soybean oil (SB10) contained vitamin A (1.32 mg/100 g), slightly below the minimum range. Nevertheless, all examined brands of soybean oil were discovered to be fortified. The results of this study align with the findings of Lisa et al. [25] and Ahmed et al. [26], indicating that fortified edible oils contained vitamin A levels below the recommended amount.

Fig. 1 (a) also showed that sunflower and palm oil brands did not comply with BSTI vitamin A standards. Among the sunflower brands, the highest and lowest amount of vitamin A was found in SF1 (1.34 mg/100 g) and SF2 (0.92 mg/100 g) respectively. Whereas palm oil sample PM1 had a higher vitamin A content (1.31 mg/100 g) than PM2 (0.99 mg/100 g). Based on the results of vitamin A [Fig. 1 (b)], it is evident that 73 % of the oil brands were below the minimum standard set by BSTI, whereas 27 % were fortified well above the minimum standard, which is also comparable with the findings of GAIN and ‘ICDDR'B [27], where they discovered that 39 % of brands were not fortified at all, 42 % of oils were fortified beyond the necessary threshold, and 24 % of brands were below the minimal standard.

### 3.2 Chemical properties of fortified edible oils

#### 3.2.1 Acid value

**Fig. 2:**
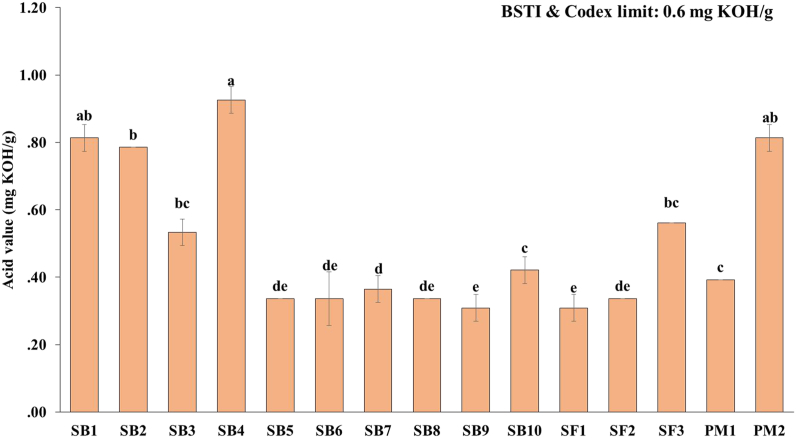
Acid value of fortified edible oils (values are mean ± SD of three replicates); SB: soybean, SF: sunflower, PM: palm; Bar graphs with different letters denote a significant difference (p < 0.05).

Among all the edible oil samples highest acid value was found in soybean oil samples SB4 (0.93 mg KOH/g) whereas similar lowest acid value (0.31 mg KOH/g) was found in soybean (SB9) and sunflower oil samples (SF1). Different soybean oil brand's (SB) acid values ranged between 0.31 and 0.93 mg KOH/g (Fig. 2). The mean acid values for soybean oil brands differed significantly (*p* < 0.05).

From Fig. 2, among the sunflower oil brands, SF1 and SF3 possessed the lowest and highest acid values of 0.31 and 0.56 mg KOH/g, respectively. The values were also within the acceptable limits (0.6 mg KOH/g). When it comes to palm oil samples, the PM2 oil sample, which differs significantly (*p* < 0.05) from the PM1 sample, has the highest acid value (0.81 mg KOH/g). Variations in acid value among oil samples can be attributed to differences in moisture concentrations, as well as distinct refining and deodorization processes [28]. The acid values of the edible oils in this investigation were comparable to the findings of [29]. From Fig. 2, it was revealed that four edible oil brands (SB1, SB2, SB4, and PM2) had acid values slightly more than the allowable limit (maximum 0.6 mg KOH/g). These higher acid levels indicate that oil triglycerides may be transformed into fatty acids and glycerol, which may promote the rancidity of the oil to a larger extent [30]. Most of the tested oil samples' lowest acid values revealed that the triacylglycerols had not been hydrolyzed, which indicates good storage stability [31].

#### 3.2.2 Free fatty acids

**Fig. 3:**
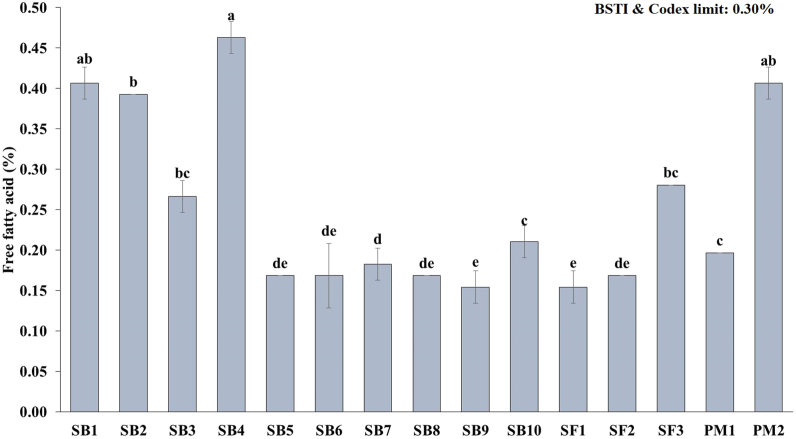
Free fatty acids of fortified edible oils (values are mean ± SD of three replicates); SB: soybean, SF: sunflower, PM: palm; Bar graphs with different letters denote a significant difference (p < 0.05).

Fig. 3 depicts the free fatty acids, a measure of the hydrolytic rancidity of the various fortified edible oils. Among the samples, soybean oil samples (SB4) recorded with highest free fatty acid value of 0.46 % and lowest value (0.15 %) were found in both SB9 and sunflower oil SF1 samples.

Among the soybean oil samples, free fatty acids ranged between 0.15 and 0.46 % which was significantly different (*p* < 0.05). The majority of the oil samples had free fatty acids contents that were less than 0.30 percent, which is the acceptable limit by both the Codex standard [32] and the BSTI standard [24] for edible oils. Three soybean oil brands, SB1 (0.41 %), SB2 (0.39 %), and SB4 (0.46 %), were found to be a little more than the standard value. The causes of high free fatty acids may be due to improper processing or distillation, the presence of microorganisms, improper storage, exposure to light, etc. [33]. However, these values were consistent with the results of Lisa et al. [25]. The free fatty acid concentration of sunflower samples was also determined and found to be within the acceptable range (<0.30 %) where SF1 and SF2 had 0.15 % and 0.17 % respectively. In contrast, palm oil sample PM2 had a higher free fatty acids (0.41 %) than the permitted limit whereas PM1 (0.20 %) was within acceptable limit. An increase in free fatty acid levels and a decrease in the overall unsaturation of oils are the two main characteristics of the oxidative and chemical changes that occur in oils during storage [34]. Therefore, the variation of free fatty acid in all samples may be due to variations in the refining and storage processes.

#### 3.2.3 Peroxide value

**Fig. 4:**
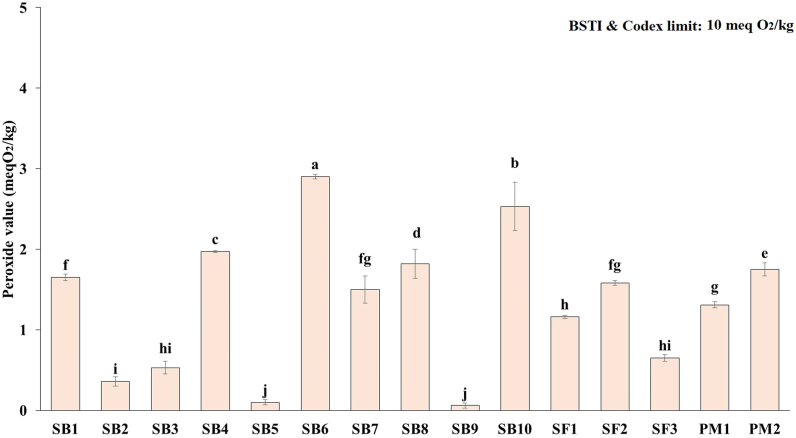
Peroxide value of fortified edible oils (values are mean ± SD of three replicates); SB: soybean, SF: sunflower, PM: palm oil; Bar graphs with different letters denote a significant difference (*p* < 0.05).

Among all the oil samples the highest and lowest peroxide value was found in soybean oil brands. The peroxide values depicted in Fig. 4 for the soybean oil samples ranged from 0.06 to 2.9 meq O_2_/kg. The highest peroxide value was observed in SB6 (2.9 meq O_2_/kg), while the lowest value was found in SB9 (0.06 meq O_2_/kg) oil samples. There were substantial differences (*p* < 0.05) between the mean peroxide values of the various brands of soybean oil.

Nevertheless, all of the samples' peroxide values fell within the permitted ranges (up to 10 meq O_2_/kg). In terms of peroxide content, SF2 (1.58 meq O_2_/kg) and SF3 (0.65 meq O_2_/kg) were the two sunflower oils with the highest and lowest values, respectively, and differed significantly (*p* < 0.05). Compared to PM1 (1.35 meq O_2_/kg), PM2 had the highest value (1.75 meq O_2_/kg) in the palm oil samples. However, all sample values fell within the permitted ranges established by Codex and BSTI [24,32]. In general, the research revealed that all the oil samples had minimum peroxide values with acceptable consumption quality. Sarwar et al. [22] and Badr et al. [35] reported similar peroxide values for fortified oil samples.

#### 3.2.4 Iodine value

**Fig. 5:**
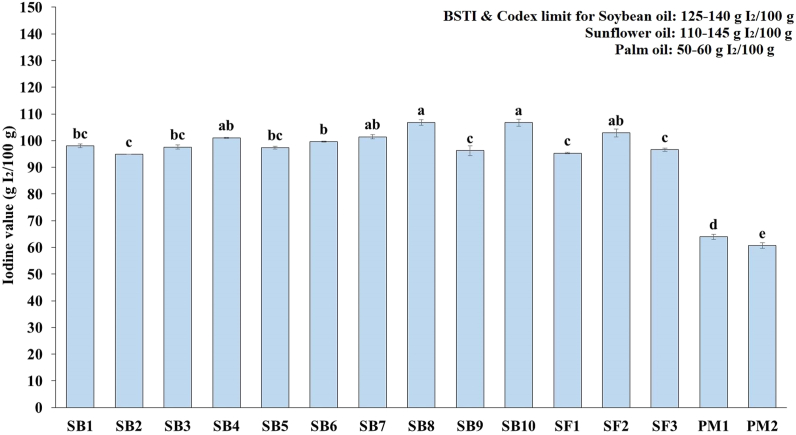
Iodine value of fortified edible oils (values are mean ± SD of three replicates); SB: soybean, SF: sunflower, PM: palm; Bar graphs with different letters denote a significant difference (p < 0.05).

The iodine values of the oil samples are depicted in Fig. 5 where the highest value was found in sunflower oil (SB8) and lowest was found in palm oil (PM2) samples. Among the soybean oil samples iodine value ranged from 94.86 to 106.79 g I_2_/100 g oil. The lowest iodine value was recorded in SB2 (94.86 g I_2_/100 g) and the highest in SB8 (106.79 g I_2_/100 g), which varied significantly (*p* < 0.05). As per results, the iodine value was observed to be lower in soybean and sunflower oils, which are more inadequate concerning the Codex standard value (125–140 I_2_/100 g for soybean oil and 110–145 g I_2_/100 g for sunflower oil) [32]. Whereas in the case of palm oil, the samples were found within the acceptable range (50–60 I_2_/100 g). Wali et al. [36] and Babatunde & Bello [37] showed comparable iodine values for edible oils, which is consistent with the findings of our investigation.

Iodine values that were noticeably higher in the SB8 and SB10 oil samples showed that the fatty acids present were mostly unsaturated, particularly in the oleic oil acid. According to earlier research, compared to palm oils, sunflower and soybean oils have more significant levels of unsaturated fatty acids [38] which are similar to our findings.

#### 3.2.5 Saponification value and glycerin content

**Fig. 6:**
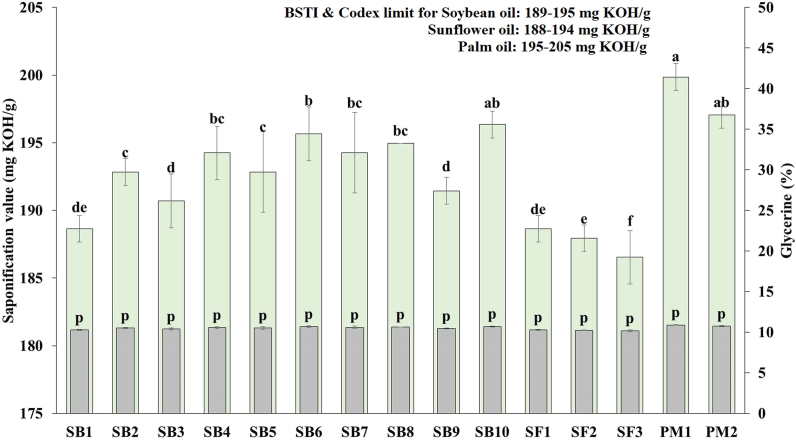
Saponification value and glycerin percentage of fortified edible oils (values are mean ± SD of three replicates); SB: soybean; SF: sunflower; PM: palm; Bar graphs with different letters denote a significant difference (*p* < 0.05).

Edible oil samples' saponification values varied from 188.64 to 196.35 mg KOH/g for soybean, 186.53–188 mg KOH/g for sunflower, and 197.05–199.86 mg KOH/g for palm oil, respectively (Fig. 6, primary axis). Whereas the glycerin content of all fortified edible oils remains more or less similar (10 %) which is depicted in the secondary axis of Fig. 6. The glycerine content was not significantly different (*p* > 0.05), which is also in agreement with the findings of Quader et al. [29] and Wali et al. [36].

Most of the results for saponification numbers met the requirements of the BSTI standards (soybean:189–195 mg KOH/g, sunflower:188–194 and palm:195–205) except sunflower oil. The mean saponification values of soybean oil brands significantly differ (*p* < 0.05) from each other's. Glycerides with the largest molecular weights were present in SB1 because it had the lowest saponification value (188.64 mg KOH/g) [39]. Higher molecular weight fatty acids in glycerides or fewer ester linkages are indicated by lower saponification values, and vice versa [40]. In contrast higher values of palm oil samples than soybean and sunflower oils indicated a high content of triacylglycerol.

### 3.3 Physical properties of fortified edible oils

#### 3.3.1 Density and specific gravity

The soybean oil samples had a density that varied from 0.855 to 0.923 g/cm³ (Table 2). The highest density (0.923 g/cm³) was found in SB5and SB2, whereas the lowest (0.855 g/cm³) was found in SB1 soybean oil, but the values were nor significantly different (*p* > 0.05). Kumar et al. [20] found similar densities for soybean oil. While in sunflower and palm oils, the density ranged from 0.913 to 0.922 g/cm³. Moreover, Koushki et al. [41] and Kumar et al. [42] showed comparable density values for palm oil and sunflower oil. It was observed that mean differences were statistically significant (*p* < 0.05). According to the findings of this research, soybean and sunflower oils’ densities were higher when compared to the density of palm oil.

**Table 2:** Physical properties of edible oils.

Brands	Density (g/cm³)	Specific gravity	pH at 30 °C	Viscosity (mPas) at 30 °C	Refractive index at 30 °C	Smoke point (°C)	Color			Odor	Rancidity
							L*	a*	b*		
SB1	0.855 ± 0.095^c^	0.85 ± 0.0942^c^	6.37 ± 0.007^b^	43.95 ± .070^bc^	1.49 ± 0.00008^a^	232	27.29 ± 0.88^a^	−0.93 ± 0.02^a^	4.13 ± 0.45^a^	Inoffensive	Nil
SB2	0.923 ± 0.0002^a^	0.917 ± 0.0004^a^	6.41 ± 0.035^b^	43.35 ± .495^bc^	1.49 ± 0.000001^a^	233	27.71 ± 0.13^a^	−0.92 ± 0.04^a^	3.99 ± 0.12^a^	Inoffensive	Nil
SB3	0.922 ± 0.0007^ab^	0.916 ± 0.0009^a^	6.64 ± 0.014^b^	49.25 ± .354^a^	1.48 ± 0.00008^a^	236	27.32 ± 0.59^a^	−1.29 ± 0.02^b^	5.36 ± 0.06^b^	Inoffensive	Nil
SB4	0.922 ± 0.0003^ab^	0.916 ± 0.0002^a^	6.04 ± 0.042^b^	44.90 ± .141^b^	1.48 ± 0.00001^a^	235	27.57 ± 0.47^a^	−1.09 ± 0.08^a^	4.52 ± 0.19^a^	Inoffensive	Nil
SB5	0.923 ± 0.0003^a^	0.918 ± 0.00005^a^	6.39 ± 0.064^b^	45.00±0^ab^	1.49 ± 0.00006^a^	238	25.67 ± 0.66^a^	−1.39 ± 0.03^b^	5.98 ± 0.19^b^	Inoffensive	Nil
SB6	0.922 ± 0.0007^ab^	0.917 ± 0.0004^a^	6.52 ± 0.014^b^	44.70 ± .141^b^	1.48 ± 0.00002^a^	233	27.39 ± 0.18^a^	−0.93 ± 0.04^a^	3.73 ± 0.11^a^	Inoffensive	Nil
SB7	0.922 ± 0.0006^ab^	0.917 ± 0.00004^a^	6.55 ± 0.057^b^	45.00±0^ab^	1.48 ± 0.00009^a^	236	26.87 ± 0.12^a^	−1.32 ± 0.08^b^	5.44 ± 0.22^b^	Inoffensive	Nil
SB8	0.922 ± 0.0005^ab^	0.917 ± 0.0002^a^	6.44 ± 0.092^b^	41.00±0^c^	1.49 ± 0.00011^b^	233	27.25 ± 0.41^a^	−1.19 ± 0.02^b^	4.78 ± 0.06^a^	Inoffensive	Nil
SB9	0.920 ± 0.00002^ab^	0.915 ± 0.0006^a^	7.08 ± 0.389^a^	44.00 ± 0^b^	1.48 ± 0.0002^a^	235	27.45 ± 0.04^a^	−0.8 ± 0.08^a^	3.28 ± 0.02^b^	Inoffensive	Nil
SB10	0.917 ± 0.0028^b^	0.911 ± 0.002^b^	6.48 ± 0.007^b^	45.00±0^ab^	1.48 ± 0.0002^b^	231	27.15 ± 0.49^a^	−1.93 ± 0.04^b^	8.88 ± 0.27^b^	Inoffensive	Nil
SF1	0.922 ± 0.00001^x^	0.916 ± 0.0006^x^	6.46 ± 0.064^x^	41.45 ± 0.78^w^	1.49 ± 0.00004^x^	232	27.56 ± 0.00^x^	−0.67 ± 0.04^y^	2.97 ± 0.04^z^	Inoffensive	Nil
SF2	0.921 ± 0.0001^x^	0.915 ± 0.0007^x^	6.43 ± 0.028^x^	44.00±0^z^	1.49 ± 0.0002^y^	233	26.29 ± 0.08^xy^	−0.57 ± 0.03^y^	2.70 ± 0.16^z^	Inoffensive	Nil
SF3	0.922 ± 0.0007^x^	0.916 ± 0.004^x^	6.45 ± 0.021^x^	54.70 ± 0.28^y^	1.49 ± 0.00007^x^	236	25.87 ± 0.4^y^	−1.08 ± 0.03^x^	4.39 ± 0.05^y^	Inoffensive	Nil
PM1	0.914 ± 0.00006^y^	0.908 ± 0.0001^y^	4.68 ± 0.141^y^	60.65 ± 0.7^x^	1.48 ± 0.0001^y^	230	26.76 ± 0.07^xy^	−1.51 ± 0.08^y^	7.65 ± 0.21^x^	Inoffensive	Nil
PM2	0.913 ± 0.0005^y^	0.908 ± 0.0004^y^	6.5 ± 0.00^x^	59.95 ± 0.35^x^	1.48 ± 0.0001^y^	228	25.94 ± 0.05^y^	−0.83 ± 0.03^x^	8.59 ± 0.08^x^	Inoffensive	Nil

*Note:* Values are mean ± SD of three replicates (except smoke point); SB: soy bean, SF: sunflower, PM: palm; values with varied superscripts in the same column are significantly different (*p* < 0.05).

In Table 2, the specific gravity of soybean oil varied between 0.85 and 0.918 at 30 °C. The SB5 and SB1 soybean oil brands had the highest and lowest values, respectively. The highest and lowest values were recorded for SB5 and SB1 oil brands. The values for soybean oil brands were not significantly different (p > 0.05). Table 1 also revealed that palm oil (0.908) had a lower specific gravity than soybean (0.855–0.923) and sunflower oil (0.915–0.916). A high concentration of linoleic acid was likely responsible for the soybean oil's highest specific gravity [43]. These results were consistent with the findings provided by Mengistie et al. [44].

#### 3.3.2 pH and viscosity

The pH ranged between 6.04 and 7.08 for soybean oil (*p* < 0.05), 6.43 and 6.46 for sunflower oil, and 4.68 and 6.5 for palm oil samples (*p* < 0.05). These values are slightly higher than the pH (4.4–5.4) values reported by Kumar et al. [42], which demonstrates that, except for palm oil sample PM1 (4.68), all of the oil samples used in this investigation are slightly acidic and nearly neutral. Among soybean oil brands, SB3 was the most viscous (49.25 mPas), whereas SB8 was the least dense (41.00 mPas). There were substantial differences (*p* < 0.05) in the viscosities of several brands of soybean oil. Compared to soybean (41.00–49.25 mPas) and sunflower oils (41.45–54.70 mPas), palm oil (59.95–60.65 mPas) was found to be more viscous. A similar higher viscosity of palm oil (61.75 mPas) was also observed by Wali et al. [36]. Oil with a high viscosity value either has a lengthy molecular chain or has substantial unsaturation [45]. It may also conclude that due to the high molecular mass, palm oil is more stable and viscous than other oils [10].

#### 3.3.3 Refractive index (RI)

The RI for soybean, sunflower, and palm oils recorded 1.48–1.49, 1.49, and 1.48, respectively, which is also somewhat higher than the threshold established by the Codex Alimentarius Commission, but comparable with findings from other studies like Koushki et al. [41] and Mengistie et al. [44]. Most soybean and palm oils had a comparatively lower RI than sunflower oil. The high refractive index (1.49) of sunflower and four soybean oil brands suggests that the fatty acids in this oil may be more unsaturated or have a more significant concentration of carbon atoms than those in palm oil [46].

#### 3.3.4 Smoke point

When choosing oil for frying, one of the key considerations is its smoke point [47]. As shown in Table 1, smoke points ranged from 231 to 238 °C, 232–236 °C, and 228–230 °C for soybean, sunflower, and palm oils, respectively. According to the results, the SB5 found itself more thermally stable. Whereas the palm oil samples were attributed to low thermal stability. Other samples' smoke points were more in line with the limits permitted by the Codex Alimentarius Commission [48]. Nevertheless, many oil samples in this study showed higher smoke points than recommended but agreed with the smoke points examined by Sarwar et al. [22].

#### 3.3.5 Color

The color numbers are coordinates in a 3-dimensional color space. From Table 2, SB2 (L = 27.71), SF1 (L = 27.56), and PM1 (L = 26.76) demonstrated maximum lightness compared to the other oil samples. Where SB10 (a* = −1.93), SB5 (a* = −1.08), and PM1 (a* = −1.51) are attributed with the maximum greenish appearance. SB10 (b* = 8.88), SF3 (b* = 4.39), and PM2 (b* = 8.59) exhibited a more pronounced reddish color compared to the other oil samples. It is likely that inadequate color reduction during the bleaching accounted for the various appearances of the oil samples. Furthermore, oils may become colored due to excessive heating, dilution ratios, the presence of soluble contaminants, and other unfinished compounds [47].

#### 3.3.6 Odor and rancidity

The edible oil samples' odor was analyzed and recorded as inoffensive and satisfactory. There was no evidence of rancidity in any of the samples because of storing in a cold environment and shielded from light and air.

### 3.4 Correlation analysis

**Fig. 7:**
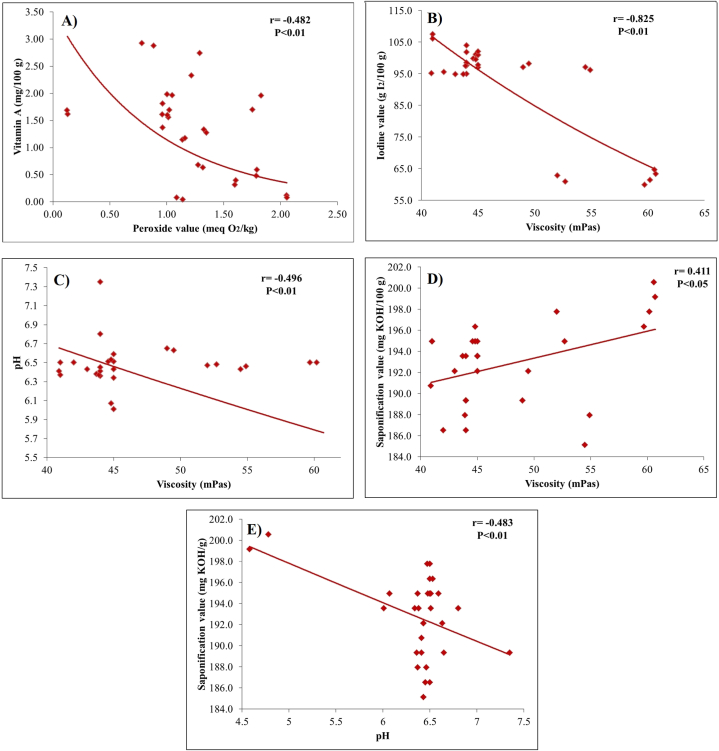
Correlation analysis between: A) Vitamin A and peroxide value; B) Iodine value and Viscosity; C) pH and Viscosity; D) Saponification value and viscosity; E) Saponification value and pH.

The peroxide values and vitamin A content of the various oil samples were shown to be significantly and inversely correlated (*p* < 0.01) [Fig. 7 (A)]. Pearson correlation analysis revealed that there is a moderate negative correlation between vitamin A content of edible oils with peroxidation value. Retinyl palmitate may be able to scavenge free radicals; hence the significant loss of vitamin A is likely related to the advancement of lipid oxidation. According to the current study, retinyl palmitate stability and lipid oxidation are significantly correlated (r = −0.482, *p* < 0.01). A similar correlation was observed by Pignitter et al. [49], where vitamin A (retinyl palmitate) showed a negative correlation (r = −0.930, *p* < 0.001) with the peroxide value of edible oils. From pearson correlation, it was revealed that chemical parameters such as acid value (r = −0.301, *p* > 0.05), saponification value (r = 0.143, *p* > 0.05), iodine value (r = 0.036., *p* > 0.05) showed non-significant (*p* > 0.05) correlation with vitamin A content.

The linear relationship between viscosity and iodine value [Fig. 7 (B)] demonstrated that viscosity decreased as iodine index increases, indicating that oils with higher percentages of unsaturated fatty acids have lower viscosities [50]. This may be because this oil contains more unsaturated fatty acids with a lower melting point and more fluid at room temperature. Oils with a higher degree of unsaturation usually have more double bonds in their fatty acid chains, which introduce kinks in the fatty acid chains, preventing the molecule from packing tightly together and contributing to lower viscosity [20]. A significant negative correlation (*p* < 0.01) has been observed between vegetable oil's viscosity and pH [Fig. 7 (C)]. It demonstrated that at lower pH vegetable oils might undergo hydrolysis, which can cause a significant (*p* < 0.01) increase in viscosity due to the formation of free fatty acids [51]. In contrast, oil with higher pH may undergo saponification with lower saponification value [Fig. 7 (E)]. Whereas positive correlation (*p* < 0.05) was observed between saponification values and viscosity of the tested oil samples [Fig. 7 (D)].

## 4 Conclusion

The study concludes that while all examined edible oil brands were vitamin A fortified, the majority fell below the national BSTI standard (1.5–3.0 mg/100 g). Chemical properties, including peroxide value, acid value, and free fatty acid contents, primarily determine edible oil quality. Most samples met BSTI and Codex standards for acid value, free fatty acid, and peroxide value. A noteworthy finding is the negative and moderate correlation between peroxide value and vitamin A content, suggesting that proper storage conditions are crucial to prevent oxidation. Although other chemical parameters didn't significantly correlate with vitamin A, various chemical and physical parameters showed significant correlations among themselves. In terms of physical quality parameters, most oil samples met BSTI and Codex standards. The study's novelty lies in providing concrete evidence of the inadequate presence of vitamin A in fortified oils, emphasizing the need for proper monitoring to ensure fortification and stability during transport and storage. Furthermore, enhancing the vitamin A content in fortified edible oils requires a multifaceted approach. Involving advanced fortification techniques (addition of chemical compound e.g. antioxidants), optimizing formulations to ensure the efficient delivery and retention of vitamin A, implementing stringent storage practices (nitrogen blanketing, dark packaging), and establishing rigorous monitoring and quality control measures.

## Funding statement

The authors would like to acknowledge the Ministry of Education, The Government of the People's Republic of Bangladesh for their financial assistance in the research project entitled “Assessment of stability of vitamin A in fortified edible oils and rice during prolong storage and heating (PCN Number: LS 2017583),” under which this study had been conducted; Ministry of Education, Government of the People's Republic of Bangladesh.

## Ethical approval

This study does not involve any human or animal testing.

## Data availability statement

Data will be made available on request.

## CRediT authorship contribution statement

**Rokeya Begum:** Writing – review & editing, Supervision, Resources, Project administration, Methodology, Funding acquisition, Conceptualization. **MdRakibul Hasan:** Writing – original draft, Methodology, Investigation, Formal analysis, Data curation. **Shamoli Akter:** Writing – review & editing, Methodology, Formal analysis. **MdNannur Rahman:** Writing – review & editing, Methodology, Investigation, Data curation.

## Declaration of competing interest

The authors declare the following financial interests/personal relationships which may be considered as potential competing interests:Dr. Rokeya Begum reports financial support was provided by Ministry of Education of the People's Republic of Bangladesh.
